# Lapdoctor: Multicentre Validation of a Scoring System for Preoperative Evaluation of Difficulty of Laparoscopic Donor Nephrectomy

**DOI:** 10.3389/ti.2025.14100

**Published:** 2025-04-23

**Authors:** Jacopo Romagnoli, Gionata Spagnoletti, Francesco Emilio Rossini, Roberto Iezzi, Alessandro Posa, Maria Paola Salerno, Patrizia Silvestri, Aldo Eugenio Rossini, Cristina Silvestre, Barbara Franchin, Alessandro Giacomoni, Leonardo Centonze, Marco Spada, Maurizio Iaria, Carmelo Puliatti, Lucrezia Furian

**Affiliations:** ^1^ Renal Transplant Unit, Fondazione Policlinico Universitario A. Gemelli IRCCS, Rome, Italy; ^2^ Ospedale Pediatrico Bambino Gesù IRCCS, Division of Hepatobiliopancreatic Surgery, Liver and Kidney Transplantation, Rome, Italy; ^3^ Department of Diagnostic Imaging, Oncological Radiotherapy and Hematology, Fondazione Policlinico Universitario A. Gemelli IRCCS, Rome, Italy; ^4^ Facoltà Di Medicina E Chirurgia, Università Cattolica del Sacro Cuore, Rome, Italy; ^5^ Kidney and Pancreas Transplantation Unit, Azienda Ospedaliera Universitaria di Padova, Padova, Italy; ^6^ ASST Grande Ospedale Metropolitano Niguarda, General Surgery and Transplantation, Milano, Italy; ^7^ Clinical and Experimental Medicine PhD Program, University of Modena and Reggio Emilia, Modena, Italy; ^8^ Department of General and Specialized Surgery, Division of General Surgery, Parma University Hospital, Parma, Italy

**Keywords:** laparoscopic donor nephrectomy score LDKT, living donor nephrectomy, minimal invasive, risk assessment, precision medicine

## Abstract

We previously developed and validated LAPDOCTOR (LAParoscopic-Donor-nephreCTomy-scORe), a novel scoring system for the preoperative assessment of the difficulty of living donor nephrectomy (LDN). To prove its significance, we extended our investigation to a prospective, multicenter, national study. Difficulty was assessed by the operating surgeon using a scale from 1 to 3 (1-standard, 2-moderately difficult, 3-very difficult) based on eight parameters: availability of laparoscopic space, mobilization of the colon, kidney, gonadal, adrenal and renal vein, renal artery, and ureter. Donor CT-scans were blindly reviewed by a radiologist, and the LAPDOCTOR scores were compared with the difficulty levels assigned by the surgeon to investigate the match rates. One hundred eighty-five donors were enrolled, with a mean age of 54 years (range 24–77), BMI 25 kg/m2 (range 17–35), and male/female 59/126. LDN was blindly scored as standard in 45% of the cases, moderately-difficult in 52%, and very-difficult in 3%. The agreement between the LAPDOCTOR and expert donor surgeons’ rate in categorizing LDN into risk groups had a QWK of 0.711 (95% CI 0.577–0.844) with p < 0.001. The LAPDOCTOR enables precise preoperative determination of the difficulty of LDN, particularly in very difficult cases, and assessment of surgical risk in living kidney donors.

**Clinical Trial Notation:**

https://ClinicalTrials.gov, Identifier NCT05769686.

## Graphical Abstract

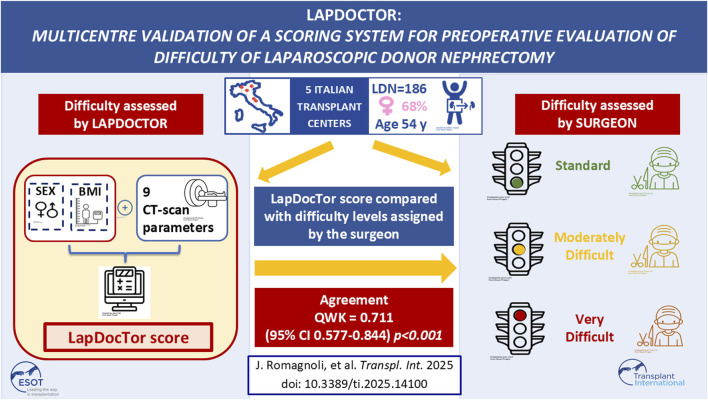

## Introduction

The superior results achieved with kidney transplantation from living donors (LDKT) have led to an increase in this method of transplantation [1]. Laparoscopic donor nephrectomy (LDN) has been spreading rapidly since it was first described in 1995 by Ratner et al. [2] introduced the principles of minimally invasive surgery in the transplantation world [3]. A part of the increase in the number of LDKT cases worldwide can be attributed to the advent of this technique [4]. LDN [5] has progressively replaced open nephrectomy owing to favorable short-term outcomes, such as less pain, reduced blood loss, and improved recovery time, and is currently the standard procedure for the procurement of kidneys from living donors [6].

It is a technically complex operation, and many surgeons prefer to select the least challenging cases, especially in the initial phase of their learning curve [7]. To make it easier, hand assistance (HALDN) has been proposed in 1998 for the first time [8], and today is widely used in many transplant centers. However, using an easier technique does not prevent unexpected difficulties, particularly in complex cases. Donors that appeared *“easy”* even after the most accurate preoperative evaluation, may inexplicably turn into difficult cases, regardless of the surgical technique or of a completely normal preoperative CT-scan. Difficulty may depend on different factors such as operator experience, donor BMI, donor anatomy, renal vascular anomalies, laparoscopic working space, quality of tissue planes, retractability of the colon and mesocolon, and sticky perinephric fat [7–9]. Unfortunately, there are no comprehensive and reliable methods to predict this type of unpredictable operative scenario.

Several attempts have been made to develop a scoring system to predict the potential difficulty of laparoscopic surgery [10–13]. However, none of them produced a real reference standard.

We previously developed the LAParoscopic Donor nephreCTomy scORe (LAPDOCTOR) [14], a calculator that showed accuracy in detecting the preoperative difficulty level of LDN in 87 patients undergoing HALDN, by combining preoperative CT-scan parameters with demographic variables. The present study was designed for prospective multicentric validation of the LAPDOCTOR.

## Materials and Methods

This prospective multicenter observational study was approved by the Ethics Committee of the Fondazione Policlinico Universitario A. Gemelli IRCCS, Rome, Italy (FPG- 2020-2939), and conducted in accordance with the tenets of the Declaration of Helsinki. The study was registered at Clinical Trials: NCT05769686 [15]. The patients signed an informed consent form at the time of enrolment.

Five Italian transplant centers were included in this prospective multicenter national study: Fondazione Policlinico Universitario A. Gemelli-Rome, Azienda Ospedaliera Universitaria - Padova, AAST Grande Ospedale Metropolitano Niguarda-Milano, Ospedale Universitario - Parma, and Ospedale Pediatrico Bambino Gesù IRCCS - Roma.

Data were collected prospectively at the participating centers and shared with the coordinating center. Radiological analysis of the preoperative CT-scans was conducted at the coordinating center.

Donors were considered eligible for the process if they met the KDIGO criteria for living kidney donation [16].

### Inclusion Criteria

Donors aged ≥18 years were deemed suitable at the end of the workup for living kidney donation.

### Exclusion Criteria

The main contraindications to kidney donation for transplantation were as follows: age less than 18 years, inability to provide consent for donation, evidence of coercion, drug abuse, evidence of malignant neoplasia, pregnancy, major respiratory or cardiovascular complications, diabetes mellitus, kidney diseases, systemic diseases with renal involvement, thrombophilia, obesity, BMI greater than 35 kg/m^2^, active infections, infections with hepatitis B, hepatitis C, and HIV, and hypertension under treatment with organ damage.

### Collected Data

The following donor data were collected: age, sex, BMI, relationship between donor and recipient, technique of LDN (pure laparoscopic, hand-assisted, or robotic), side of LDN (right or left kidney), operative time, blood loss (need for transfusion support), conversion rate, number of renal arteries, number of renal veins, incidence of postoperative major complications (Clavien-Dindo grade ≥ III), and post-operative length of stay (LOS).

### Primary Endpoint

The objective of this multicenter observational study was to validate the LAPDOCTOR, a new scoring system for preoperative prediction of the difficulty of LDN for living kidney donation in the context of transplantation.

The LAPDOCTOR is based on the analysis of 11 demographic and anatomo-radiological donor parameters, which showed a statistically significant correlation with the surgical difficulty reported by the operator in a previously conducted univariate analysis [14]. For each parameter, a progressive score was assigned based on the observed increase in difficulty. The sum of the scores assigned to each parameter produces a final score (min 11–max 33), which allocates the donor to one of three classes of progressive risk: low = 11–18, medium = 19–25, high = 26–33. The calculations were performed using a program created in Microsoft Excel (*LapDocTor calculator,* Supplementary Material S1, S2).

The validity of the objective score was evaluated by studying its correlation with the subjective judgment of the operator. This judgment was formulated based on a score (from 1 to 3) assigned by the donor surgeon to each of the following eight phases of the operation: mobilization of the colon, kidney, gonadal vein, adrenal vein, renal vein, renal artery, and ureter. The obtained score (range 8–24) allocates the donor into one of three difficulty classes (*standard*, *moderately difficult, very-difficult*).

**FIGURE 1:**
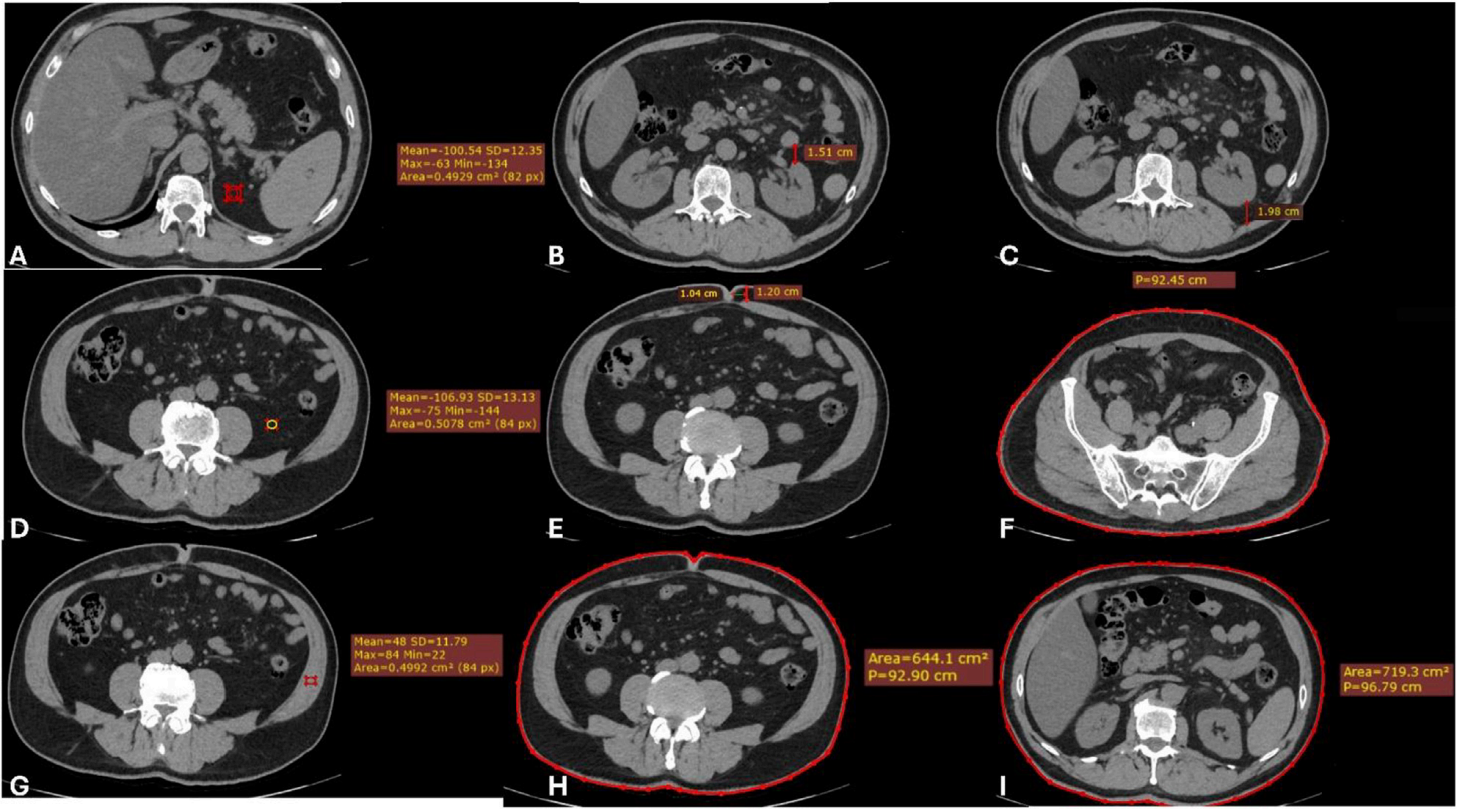
Axial CT images showing the radiological parameters considered. **(A)** Upper renal fat tissue density (just above the kidney, ROI of 0.5 cm2). **(B)** Pre-renal fat tissue thickness (at the middle third of the kidney, from the kidney to the bowel). **(C)** Retro-renal fat tissue thickness (at the middle third of the kidney, from the kidney to the muscle). **(D)** Lower renal fat tissue density (just below the kidney, ROI of 0.5 cm2). **(E)** Abdominal wall fat tissue thickness (at 1 cm from the navel). **(F)** Abdominal circumference (at the antero-superior iliac spine). **(G)** Oblique muscles density (ROI of 0.5 cm2). **(H)** Abdominal circumference (at the navel). **(I)** Abdominal circumference (at the 12th rib).

All preoperative unenhanced and contrast-enhanced CT-scans were blindly reviewed by a radiologist, recording the following parameters: renal artery and vein number and anatomical variants, abdominal circumference (measured at the 12th rib, umbilicus, and iliac bone), pre- and post-renal visceral fat thickness and density on the side of the procured kidney, periumbilical subcutaneous fat tissue thickness, and oblique muscle density. Density was measured in Hounsfield Units (HU) on unenhanced CT-scans using a circular region of interest (ROI) with a radius of 5 mm to evaluate the median measured value [14] (Figure 1).

The CT-scans have been collected and evaluated retrospectively in order to keep the blindness of the surgeons at the time of the intervention.

In the present study, we explored the correlation between LAPDOCTOR scores and difficulty levels assigned by the operating surgeon in a multicenter setting. All surgeries were performed by one surgeon per center.

### Statistical Analysis

Statistical analysis was based on examining the inter-rater reliability or agreement between the two scores (preoperative objective and postoperative subjective scores obtained from the operator) using quadratically weighted (QWK) Cohen’s Kappa and corresponding 95% confidence intervals (CIs). A kappa of <0.00 is considered poor agreement, 0.00–0.20 slight agreement, 0.21–0.40 fair agreement, 0.41–0.60 moderate agreement, 0.61–0.80 substantial agreement, and 0.81–1.00 almost perfect agreement [17]. Moreover, according to Fleiss interpretation [18] values, a Kappa greater than 0.75 may be taken to represent excellent agreement beyond chance. Continuous and normally distributed variables are expressed as mean ± standard deviation, and categorical data are expressed as proportions. Data were recorded using Excel 2016 (Microsoft Corporation, Redmond, Washington, DC, United States) and analyzed using SPSS 25.0 (IBM Corporation, Armonk, New York, NY, United States).

## Results

During the study period, 185 donors from five italian transplant centers were enrolled. The patient demographics are shown in Table 1.

**TABLE 1:** Characteristics of participants.

Donors, n	185
Age, years	53.5 (10.6)
Male	59 (32%)
Female	126 (68%)
BMI, kg/m^2^	25.1 (3.6)
Related	111 (60%)
ABO incompatible	29 (16%)
Nephrectomy Side [Left/Right]	166/19 (90%–10%)
Renal vascular Anomalies	33 (18%)
Multiple arteries	30 (16%)
Surgical Technique	
Hand-assisted	75 (41%)
Pure Laparoscopic	69 (37%)
Robotic	41 (22%)

Data are mean (SD) or n (%).

The mean age of donors was 54 years (range 24–77 years), 126/185 donors (68%) were female, and 111/185 (60%) were related to the recipient. Twenty-nine donors (16%) were ABO incompatible. The mean BMI was 25 kg/m^2^ (range, 17–35).

The technical approach varied among centers: in 75/185 cases (41%), LDN was performed using a hand-assisted approach; in 69 cases (37%), using a pure laparoscopic approach; and in 41 cases (22%), using a robotic approach.

The left kidney was preferred in 166/185 cases (90%), whereas the right kidney was retrieved in only 19/185 cases (10%). Among the right kidney procedures (19, 10%), the majority were performed using a hand-assisted approach (11/19, 57%), which seems to make transplant surgeons feel more confident in recovering the right kidneys [19] and a robotic approach in approximately one-third of the cases (6/19, 31%). This approach was chosen because it is the routine technique used for both the right and left kidneys in one of the five participating centers.

Regarding anatomical variations, 33 kidneys (18%) had vascular anatomical variants, with the majority (30 cases, 16%) presenting with multiple arteries.

The mean operative time (from skin incision to skin closure) was 267 ± 79 min, with a mean laparoscopic time of 209 ± 86 min. The operative time was longer for hand-assisted procedures than for laparoscopic or robotic procedures (data shown in Table 2).

**TABLE 2:** Results of LDN.

Number of procedures	185
Operative Time, minutes	267 (79)
Hand-assisted	289 (58)
Pure Laparoscopic	245 (87)
Robotic	266 (89)
Laparoscopic Time (minutes, mean ± standard deviation)	209 (86)
Hand-assisted	232 (56)
Pure Laparoscopic	213 (104)
Robotic	162 (96)
Conversion, n	1 (0.5%)
Complications according to Clavien-Dindo, n	19 (10.2%)
Grade I	6 (3.2%)
Grade II	9 (4.9%)
Grade III a-b	3 (1.6%)
Length of stay	5 (2)

Data are mean (SD) or n (%).

All procedures were performed transperitoneally. There was one case (0.5%) of conversion of a left pure LDN to an open nephrectomy, which resulted in a successful operation, preserving both patient and graft survival.

The overall incidence of complications was 10.2%, which is consistent with the literature (8%–18%) [5]. According to the Clavien-Dindo classification, only 1.6% were grade III (a-b) and 4.9% were grade II (Table 2).

After all procedures, the first operator collected a survey, grading each of the eight steps from 1 to 3 based on the level of perceived difficulty. The procedures were classified as *standard* in 83/185 cases (45%), *moderately difficult* in 97/185 (52%), and *very difficult* in 5/185 (3%).

In Supplementary Table S4, we reported values of cases stratified as standard, moderately difficult, and very difficult, further categorized by surgical phase for each surgeon.

A single radiologist blindly reviewed all pre-operative CT-Scan images and collected anatomical and radiological donor parameters. Based on these parameters, BMI and sex were added (Supplementary Table S3). The LAPDOCTOR classified 83/185 procedures (45%) as *standard*, 97/185 (52%) as *moderately difficult*, and 5/185 (3%) as *very difficult*.

**FIGURE 2:**
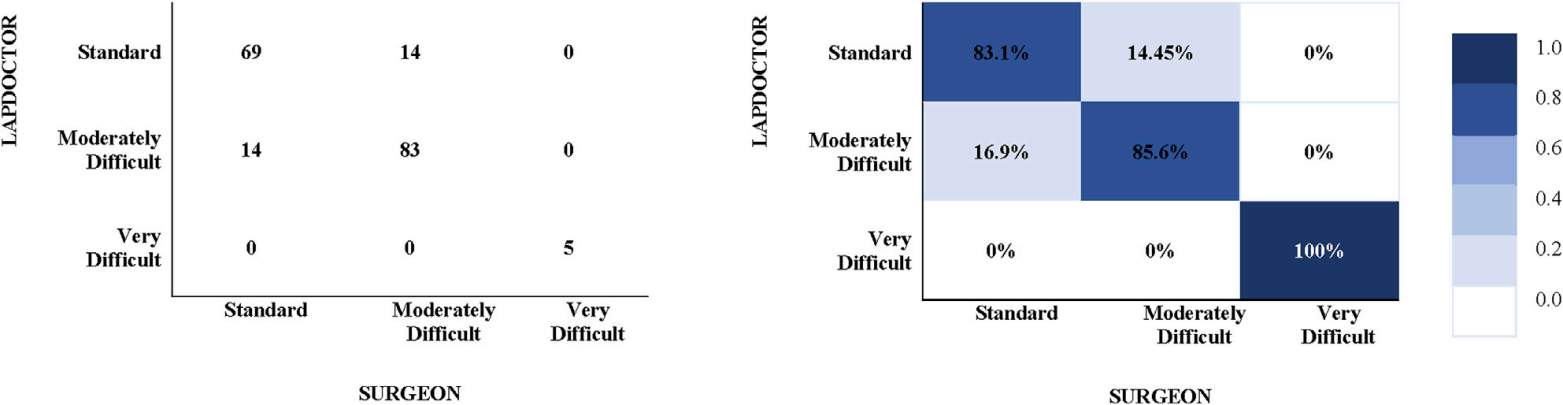
Agreement rate for level of difficulty between LAPDOCTOR score and Donor Surgeon’s score: excellent concordance in risk group classification by a QWK of 0.711 (95% CI 0.577–0.844), p < 001.

All data were centrally resumed in the dataset. The agreement between LAPDOCTOR and the donor surgeons’ rate in categorizing LDN into *standards*, *moderately difficult*, and *very difficult* risk groups had a QWK of 0.711 (95% CI 0.577–0.844) with p < 0.001 (Figure 2). Considering the individual QWK, “*standard*” cases had a QWK of 0.831 (95% CI, 0.550–0.838, p < 0.001), *moderately difficult* 0.856 (95% CI, 0.552–0.841, p < 0.001), and *very difficult* 1.00 (95% CI, 0.856–1.144, p < 0.001).

We performed a sub-analysis of cases with observed discrepancy between the surgeon’s judgment and the LAPDOCTOR prediction and found that in cases deemed *standard* by the surgeon but *moderately difficult* by LAPDOCTOR, the average values of most parameters tended to align more closely with those of the *moderately difficult* LAPDOCTOR cases. We speculate that the greater confidence of an experienced surgeon may have resulted in an easier perception of *moderately difficult* cases.

A similar consideration applies to cases where the surgeon’s experience of a *moderately difficult* operation did not match the LAPDOCTOR’s “*standard*” rating.

## Discussion

Our study introduces a novel difficulty scoring system for LDN that enables preoperative identification of technically challenging cases based on readily available donor parameters. By analyzing 185 living donors within the context of a multicenter prospective clinical trial, we demonstrated that this grading system can accurately identify potentially difficult donors and define the expected level of difficulty, regardless of the type of laparoscopic approach used.

The implications of this study are significant. In the presence of multiple potential donors, the LAPDOCTOR can assist in selecting the least challenging donor. Conversely, if only one donor is available, it can help the surgeon plan a safer operation by being aware of potential difficulties. From a training perspective, it allows for the selection of easier cases for junior fellows, thereby reducing unnecessary risks to the donor, surgeon, and trainee.

This study was conducted in response to the strong need for tools that help donor surgeons plan safer living donor operations. Several difficulty scoring systems have been proposed for laparoscopic surgery [10–13], with models based on preoperative donor characteristics or preoperative imaging, however, we did not find comparable methods to comprehensively and reliably assess difficulty of LDN. Surgeons have also developed renal morphometry scoring systems, such as the R.E.N.A.L. nephrometry score, PADUA prediction score, and centrality index (C-index), to analyze anatomical findings that can predict the complexity of nephrectomy and the likelihood of complications [20–22]. The Mayo group proposed the Mayo Adhesive Probability Score (MAP) [23] to predict the presence of adherent perinephric fat. Other scoring systems have used various variables, particularly radiographic variables, to correlate the operative difficulty and postoperative outcomes [24].

Most studies have used factors such as sex, body mass index (BMI), perirenal fat, and number of renal arteries and veins as measures of difficulty. Ratner et al. [7] attempted to create a scoring system to determine whether anatomical or radiologic parameters could accurately assess the technical difficulty of LDN preoperatively. They reviewed CT scans and graded the different phases of the operation on a scale of 1–4 but found that technical difficulty could not be predicted by body habitus from the variables examined in their study.

However, none of these scoring systems have considered a multiparametric approach or combined objective preoperative data with an intraoperative surgeon’s score based on perceived difficulty. To overcome the bias of subjectivity, we designed a multicenter study involving five experienced transplant surgeons from five major Italian transplant centers. In three centers, LDN was performed using different laparoscopic approaches (pure laparoscopy or robotic) based on the surgeon’s experience. In the remaining two centers HALDN was the standard.

This could be a limitation of our study; however, LAPDOCTOR compared the difficulty of different donors using the same set of parameters, regardless of the approach. The term of comparison used to validate the scoring system is the experience of the operating surgeon with the technique with which they are most confident. The LAPDOCTOR has shown no statistical differences in its ability to identify difficult cases in donors operated with either hand-assisted, pure laparoscopic, or robotic techniques. Notably, there was a full match for the very difficult cases. Nonetheless, right now, whether there is a more favorable technique cannot be drawn neither from our data, nor from literature’s data.

LAPDOCTOR proved helpful in our practice for the preoperative surgical evaluation of living donors. With a simple excel sheet saved on the PC desktop of the transplant clinic, ready to be filled with a set of easy to obtain parameters, even a junior surgeon can objectively categorize the surgical risk of LDN, instead of relying on subjective judgment “by eye,” based only on personal experience of a senior surgeon. Moreover, in the setting of an academic training center, the utility of LAPDOCTOR resides in its ability to sort out the most adequate cases to train transplant fellows in this very delicate operation. In many centers part of this operation is entrusted to senior trainees, under consultant’s supervision and LAPDOCTOR facilitates the choice of the proportion of risk one can decide to allocate them, depending on the individual skills and experience of each trainee. Of note, the longer operation times observed in donors operated with HALDN are indeed easily explained by the training needs, one of the main reasons for the choice of this technique being the possibility to allow trainees to make experience and progress with this operation, while preserving donor safety and senior surgeon’s coronaries. The dissemination of LAPDOCTOR, by standardizing the scoring system, would also help in the mutual exchange and interpretation of collected data coming from different centers, thus promoting further progress in our knowledge of such a sensitive topic.

### Limitation

The present study has some limitations that need to be acknowledged. The study is multi-centric, but all participating centers were from a single country (Italy); we included different surgical techniques, and the sample is relatively small, so that a sub-group analysis is not feasible and does not allow for the individual validation of LapDocTor. Since our main purpose was to challenge the ability of the scoring system to predict difficulty, we did not assess long-term outcomes. Despite the excellent agreement between our score and the surgeon’s judgment, the latter remains inherently subjective and may explain the discordance found for some cases, likely due to individual surgeon’s experience.

For these reasons, our findings will require external validation in a larger, specifically designed, possibily multi-ethnic, international cohort study.

### Conclusion

The LAPDOCTOR is a very simple scoring system that accurately determines the expected level of difficulty for laparoscopic donor nephrectomy by utilizing donor demographics and CT scan parameters. It is particularly effective in identifying the most challenging cases, enabling surgeons to plan operations more safely by being aware of the potential risks. Additionally, it is valuable for training purposes as it assists in selecting easier cases for surgical training, thereby minimizing unnecessary risks for the donor, surgeon, and trainee.

Further studies are warranted to investigate the correlation between the LAPDOCTOR scores and long-term patient and graft outcomes.

## Data Availability

The raw data supporting the conclusions of this article will be made available by the authors, without undue reservation.

## Supplementary Material

SUPPLEMENTARY FIGURE S1 Examples of results calculated with LapDocTor.

## Abbreviations

BMI, body mass index; HALDN, hand assistance laparoscopic donor nephrectomy; LDN, living donor nephrectomy; LAPDOCTOR, LAParoscopic DOnor nephreCTomy score; LDKT, kidney transplantation from living donors; LOS, length of stay.
