# Supplementary figures and images for: Lapdoctor: Multicentre Validation of a Scoring System for Preoperative Evaluation of Difficulty of Laparoscopic Donor Nephrectomy
Source: Transpl Int. 2025 Apr 23;38:14100. doi: 10.3389/ti.2025.14100 (PMC12055550; doi:10.3389/ti.2025.14100)

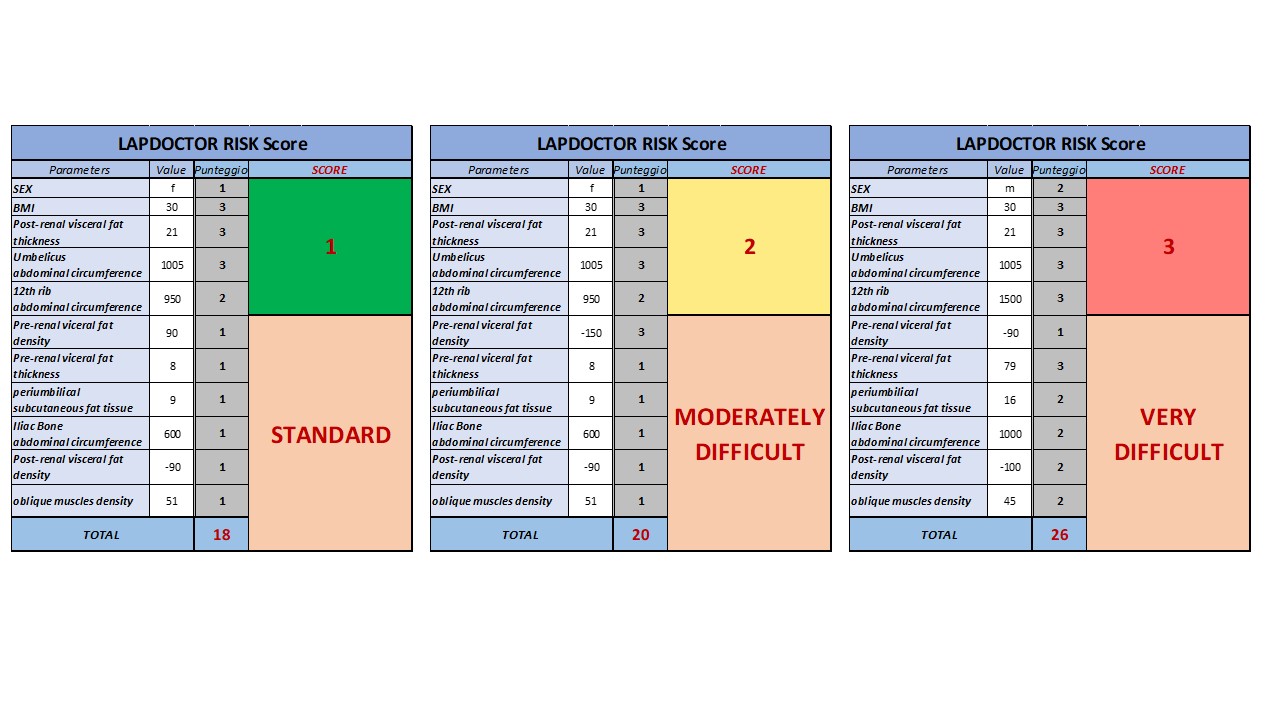

Supplement: Supplementary file 1 [file Image1.JPEG]
