# Supplementary material for: Lapdoctor: Multicentre Validation of a Scoring System for Preoperative Evaluation of Difficulty of Laparoscopic Donor Nephrectomy
Source: Transpl Int. 2025 Apr 23;38:14100. doi: 10.3389/ti.2025.14100 (PMC12055550; doi:10.3389/ti.2025.14100)
Supplement: Supplementary file 2 [file Table3.docx]

| **Table 3 LAPDOCTOR Donor CT Scan parameters** | | | | | |
| --- | --- | --- | --- | --- | --- |
| ***Variable*** | ***Mean*** | ***Standard deviation*** | ***Median*** | ***Min*** | ***Max*** |
| ***12th rib***  ***abdominal circumference*** | 904.3 | 110.2 | 903.1 | 100.4 | 1175.4 |
| ***Umbelicus***  ***abdominal circumference*** | 938.6 | 105.5 | 940.9 | 683.8 | 1232.2 |
| ***Iliac Bone***  ***abdominal circumference*** | 970.0 | 112.5 | 967.4 | 106.3 | 1268.7 |
| ***Pre-renal viceral fat thickness*** | 7.0 | 4.5 | 5.9 | 0.01 | 26.3 |
| ***Post-renal visceral fat thickness*** | 11.6 | 7.0 | 10.0 | 0.01 | 40.2 |
| ***periumbilical subcutaneous fat tissue thickness*** | 23.8 | 8.9 | 24.7 | 1.5 | 48.4 |
| ***Pre-renal viceral fat density*** | -95.0 | 13.3 | -96.0 | -122.0 | -50.0 |
| ***Post-renal visceral fat density*** | -97.3 | 19.1 | -100.0 | -126.0 | 111.6 |
| ***oblique muscles density*** | 47.9 | 9.12 | 48.0 | 16.6 | 96.9 |
| *Density was measured in Hounsfield Units (HU) on unenhanced TC scans, using a circular ROI (region of interest) with a radius of 5mm, evaluating the median measured value; the circumference and thickness of the tissue were measured in millimeters.* | | | | | |
