# Supplementary material for: Lapdoctor: Multicentre Validation of a Scoring System for Preoperative Evaluation of Difficulty of Laparoscopic Donor Nephrectomy
Source: Transpl Int. 2025 Apr 23;38:14100. doi: 10.3389/ti.2025.14100 (PMC12055550; doi:10.3389/ti.2025.14100)
Supplement: Supplementary file 4 [file Table4.docx]

| **Table S4** **Details of the operating surgeon's assessments for each study center involved** | | | | | |  |
| --- | --- | --- | --- | --- | --- | --- |
| ***SURGICAL PHASE*** |  | ***A*** | ***B*** | ***C*** | ***D*** | ***E*** |
|  | ***Difficulty class*** | **59*** | **56*** | **28*** | **22*** | **20*** |
| ***Laparoscopic workspace*** | *standard* | 52 (88%) | 46 | 18 | 18 | 11 |
|  | *moderately - difficult* | 5 (9%) | 6 | 8 | 4 | 6 |
|  | *very - difficult* | 2 (35%) | 4 | 2 | 0 | 3 |
| ***Colon mobilization*** | *standard* | 56 (95%) | 45 | 25 | 21 | 13 |
|  | *moderately - difficult* | 2 (3%) | 10 | 3 | 1 | 4 |
|  | *very - difficult* | 1 (2%) | 1 | 0 | 0 | 3 |
| ***Ureter mobilization*** | *standard* | 58 (98%) | 48 | 24 | 21 | 18 |
|  | *moderately - difficult* | 1 (2%) | 6 | 4 | 1 | 2 |
|  | *very - difficult* | 0 | 2 | 0 | 0 | 0 |
| ***Gonadal vein mobilization*** | *standard* | 57 (96%) | 40 | 26 | 18 | 16 |
|  | *moderately - difficult* | 1 (2%) | 14 | 2 | 4 | 4 |
|  | *very - difficult* | 1 (2%) | 2 | 0 | 0 | 0 |
| ***Kidney mobilization*** | *standard* | 47 (80%) | 42 | 26 | 16 | 8 |
|  | *moderately - difficult* | 5 (8%) | 12 | 1 | 4 | 10 |
|  | *very - difficult* | 7 (12%) | 2 | 1 | 3 | 2 |
| ***Adrenal vein mobilization*** | *standard* | 57 (97%) | 44 | 24 | 16 | 16 |
|  | *moderately - difficult* | 0 | 12 | 3 | 6 | 4 |
|  | *very - difficult* | 2 (3%) | 1 | 1 | 0 | 0 |
| ***Renal*** ***artery mobilization*** | *standard* | 54 (92%) | 33 | 17 | 10 | 12 |
|  | *moderately - difficult* | 3 (5%) | 17 | 11 | 9 | 5 |
|  | *very - difficult* | 2 (3%) | 6 | 0 | 3 | 3 |
| ***Renal vein mobilization*** | *standard* | 55 (93%) | 38 | 14 | 14 | 14 |
|  | *moderately - difficult* | 3 (5%) | 13 | 12 | 6 | 6 |
|  | *very - difficult* | 1 (2%) | 5 | 2 | 2 | 0 |
| ***All cases*** | *standard* | 43 (73%) | 22 | 7 | 7 | 3 |
|  | *moderately - difficult* | 14 (24%) | 31 | 21 | 14 | 16 |
|  | *very - difficult* | 2 (3%) | 1 | 0 | 1 | 1 |
| *Judgment formulated on a score (from 1 to 3) by the donor surgeon at the end of the procedure*  ** number of cases*  ***A*** *– Fondazione Policlinico Universitario A. Gemelli-Rome*  ***B*** *– Azienda Ospedaliera Universitaria - Padova*  ***C*** *– AAST Grande Ospedale Metropolitano Niguarda-Milano*  ***D*** *– Ospedale Pediatrico Bambino Gesù IRCCS - Roma*  ***E*** *– Ospedale Universitario – Parma* | | | | | |  |
